# *In Vivo* Study on the Pharmacological Interactions between a Chinese Herbal Formula ELP and Antiresorptive Drugs to Counteract Osteoporosis

**DOI:** 10.1155/2012/203732

**Published:** 2012-10-24

**Authors:** Chun-Hay Ko, Wing-Sum Siu, Hing-Lok Wong, Si Gao, Wai-Ting Shum, Ching-Po Lau, Sau-Wan Cheng, Jacqueline Chor-Wing Tam, Leung-Kim Hung, Kwok-Pui Fung, Clara Bik-San Lau, Quan-Bin Han, Ping-Chung Leung

**Affiliations:** ^1^Institute of Chinese Medicine, The Chinese University of Hong Kong, Shatin, New Territories, Hong Kong; ^2^State Key Laboratory of Phytochemistry and Plant Resources in West China, The Chinese University of Hong Kong, Shatin, New Territories, Hong Kong; ^3^Department of Orthopaedics and Traumatology, The Chinese University of Hong Kong, Shatin, New Territories, Hong Kong; ^4^Jockey Club Centre for Osteoporosis Care and Control, The Chinese University of Hong Kong, Shatin, New Territories, Hong Kong; ^5^School of Biomedical Sciences, The Chinese University of Hong Kong, Shatin, New Territories, Hong Kong; ^6^School of Chinese Medicine, Hong Kong Baptist University, Kowloon, Hong Kong

## Abstract

Antiresorptive drugs, alendronate and raloxifene, are effective in lowering bone mineral density (BMD) loss in postmenopausal women. However, long-term treatment may be associated with serious side effects. Our research group has recently discovered that a Chinese herbal formula, ELP, could significantly reduce BMD loss in animal and human studies. Therefore, the present study aimed to investigate the potential synergistic bone-protective effects of different herb-drug combinations using ovariectomized rats. To assess the efficacy of different combinations, the total BMD was monitored biweekly in the 8-week course of daily oral treatment. Bone microarchitecture, bone strength, and deoxypyridinoline level were also determined after 8 weeks. From our results, coadministration of ELP and raloxifene increased the total tibial BMD by 5.26% (2.5 mg/kg/day of raloxifene; *P* = 0.014) and 5.94% (0.25 mg/kg/day of raloxifene; *P* = 0.026) when compared with the respective dosage groups with raloxifene alone. Similar synergistic effects were also observed in BMD increase at distal femur (0.25 mg/kg/day; *P* = 0.001) and reduction in urinary deoxypyridinoline crosslink excretion (2.5 and 0.25 mg/kg/day; both *P* = 0.02). However, such interactions could not be observed in all alendronate-treated groups. Our data provide first evidence that ELP could synergistically enhance the therapeutic effects of raloxifene, so that the clinical dosage of raloxifene could be reduced.

## 1. Introduction

Osteoporosis is a degenerative disease characterized by low bone mass and deterioration of microarchitecture of bone, which increases bone fragility and susceptibility to fractures [1]. These fractures incur morbidity and mortality to the elderly. Osteoporosis is one of the most serious geriatric health problems in Europe and America [2, 3]. Approximately 30% of all postmenopausal women have osteoporosis in Europe and America. It is estimated that over 200 million people worldwide suffer from this disease [4]. By 2050, the worldwide incidence of osteoporotic hip fracture is projected to increase by 240% in women and 310% in men [5]. The economic costs due to osteoporotic fractures have increased tremendously in the past decade and are predicted to grow. Measures are needed to reduce the prevalence of osteoporosis and incidence of osteoporotic fractures. A number of drugs are currently thought to be effective for the prevention or treatment of osteoporosis. Alendronate and raloxifene are two of the drugs that have been extensively used. Both are effective in lowering bone mineral density (BMD) loss and reducing the risk of fractures [6]. Their antiosteoporotic actions are quite similar in inhibiting bone resorption process, resulting in the increase of the overall bone formation. However, there is increasing evidence that both drugs have the potential in causing a number of adverse effects upon long-term administration. For instance, long-term bisphosphonates treatments would lead to the osteonecrosis of the jaw [7]. Treatments with raloxifene may increase the risk of venous thrombosis [8]. Although these side effects are not common, there is still concern regarding the long-term use against the use of these antiresorptive agents. 

In the past few years, our research group discovered a number of Chinese medicines that can effectively prevent and treat osteoporosis. The herbal formula ELP, which contains three “kidney-tonifying” herbs: Epimedii Herba (E), Ligustri Lucidi Fructus (L), and Psoraleae Fructus (P) with a weight ratio of 5 : 4 : 1, has been shown to be prominent in promoting the osteogenic differentiation in rat mesenchymal stem cells by enhancing bone activities such as alkaline phosphatase activity and matrix calcium deposition [9]. In addition, this formula can inhibit the spinal BMD loss in aged ovariectomized osteopenic rats without any adverse effect [10]. Furthermore, we have completed a clinical trial and found that ELP could reduce both bone loss and hip fractures in post-menopausal women. This randomized controlled clinical trial illustrated that 44 mg/kg per day of ELP extract significantly inhibited the progressive bone loss in the spine of postmenopausal women (*n* = 75) after 12 months of herbal treatment [11]. There are also reports showing that one active compound of ELP, icariin can alleviate the bone loss in ovariectomized animals and promote the bone formation in osteoblastic UMR-106 cells [12]. Mechanistic studies proved that icariin acted with helioxanthin derivatives (other bone anabolic agents) could synergistically enhance bone formation [13]. Therefore, the antiosteoporotic actions of ELP are quite different from bisphosphonates and raloxifene. It is postulated that it stimulates bone formation rather than inhibiting bone resorption in the balance of bone metabolism. 

Osteoporosis is the result of an imbalance in bone remodeling, with higher bone resorption rate than bone formation rate. Enhancing the activity of bone-forming osteoblasts, plus reducing that of the bone-breaking osteoclasts, may help restoring the balance in bone metabolism and limiting bone loss in the development of osteoporosis [14]. Since ELP and western conventional drugs act on different cellular/molecular targets in bone metabolism, we hypothesized that synergistic effects would be generated when patients take both together. These medicines might be well combined to complement, or compensate for respective defects in each other. To that end, extensive study of the interactions between Chinese medicines and Western medicines is needed, and predictable adverse effects should be avoided. This herbal combination may enhance the overall osteoprotective effects so that the dosage of these antiresorptive drugs can be minimized. Using an ovariectomized rat model, we here investigated whether there are synergistic effects between ELP and antiresorptive drugs (which are greater than the effects of antiresorptive drugs alone) on increasing bone formation and decreasing bone resorption.

## 2. Materials and Methods

### 2.1. Chemicals and Reagents

All chemicals and reagents were purchased from Sigma (USA) unless otherwise specified. Alendronate sodium and raloxifene hydrochloride were obtained from Merck (GmbH) and Eli Lilly (USA), respectively.

### 2.2. Herbal Extraction and Characterization

Raw herbal materials were purchased from a single renowned supplier in Hong Kong. Morphological, microscopic, and chemical authentications were performed in accordance to Chinese Pharmacopoeia [15]. Herbarium voucher specimens of the tested herbs were deposited at the museum of the Institute of Chinese Medicine, the Chinese University of Hong Kong, with voucher specimen numbers as follows: 2004–2547 (E), 2004–2566 (L), and 2004–2568 (P). Raw herbal materials of Epimedii Herba, Ligustri Lucidi Fructus and Psoraleae Fructus, with a weight ratio of 5 : 4 : 1, were extracted under reflux in boiling water for 1 hour and the extraction was repeated twice. The aqueous extracts were collected and filtered. The filtrate was then concentrated under reduced pressure at 50°C and lyophilized into powder. The formula was subjected to standardization. The chemical profile was examined using the representative markers such as icariin (for Epimedii Herba), salidroside (for Ligustri Lucidi Fructus), and psoralen and isopsoralen (for Psoraleae Fructus) using liquid chromatography-mass spectrometry (LC-MS) (6530 accurate-mass Q-TOF LC/MS, Agilent Technologies, USA). ELP aqueous extract (1 mg/mL) was injected into an ACQUITY UPLC C18 column (2.1 × 100 mm id, particle size 1.7 *μ*m) (Waters, USA). A gradient elution was carried out using the following solvent systems: mobile phase A- double distilled water/formic acid (99.9/0.1; v/v); mobile phase B-acetonitrile. The linear gradient elution system was 100% A to 100% B for 30 min, following by standing at 100% A for 10 min. The flow rate was set at 0.3 mL/min. Identification of the chemical markers was carried out by comparing the retention times of unknown peaks to those of the standards with matched ionization products' size. Detection of ionization products was performed by monitoring positive ions of the combined parent and product compounds in multiple reaction monitoring mode (MRM). The theoretical m/z values of the parent and product ions [M+H]^+^ were set at 677.66 for icariin, [M+Na]^+^ 323.304 for salidroside, and [M+H]^+^ 187.16 for both psoralen and its isomer isopsoralen. The abundance of each marker in ELP extract was determined quantitatively.

### 2.3. Model Establishment and Treatment Protocol

An Animal Experimentation Ethics Approval had been obtained from the Animal Experimental Ethics Committee of the Chinese University of Hong Kong (Ref No. 09/068/MIS). Eighty-eight 3-month old female Sprague-Dawley (SD) rats were used and housed four per cage in a room maintained at 22°C with a 12-hour light-dark cycle. The rats were equally divided into groups of 8 individuals. Rats in OVX were ovariectomized bilaterally whereas those in Sham experienced sham surgery. During the experimental period, the rats were maintained on standard rodent chow that contained 0.9% calcium and 0.7% phosphate, and distilled water was available *ad libitum*. After three weeks of ovariectomy operation, ELP extracts and two antiresorptive drugs (alendronate (A) and raloxifene (R)) were orally administered to each rat intragastrically for 8 weeks, as shown in Table 1. Rats' body weight was recorded every week to assess the changes. Fasting 24-hour urine samples were collected by placing the animals in individual metabolic cages for one day before sacrifice. Urine samples were acidified with 2 mL of 1 M hydrochloric acid and centrifuged at 1200 ×g for 10 minutes at 4°C to remove contaminants, and aliquots were stored at −20°C until assayed. After sacrifice, blood samples were taken from the abdominal vena cava. Then serum samples were obtained by centrifuged at 3000 ×g for 20 minutes at 4°C and stored at −80°C before assessment of biochemical parameters. The uteruses were removed and weighed immediately. The success of ovariectomy was confirmed at necropsy by failure to detect ovarian tissue and by observation of marked atrophy of the uterine horns.

The animals were divided into 11 groups as shown in Table 1. Group 1 was sham operated, while the remaining 10 groups of rats were ovariectomized (OVX). The dose of ELP used in the experiment (0.35 g/kg/day; groups 3 and 8–11) was proven to be effective in our previous study [10]. The dose (equivalent to an adult human intake of 6–12 g crude herb) was calculated from the human equivalent dose table [16], multiplied by a factor of extraction yield. Similar justifications were also applied for A and R. Our pilot studies indicated that the optimal concentrations for A and R were 0.5 (group 4) and 2.5 mg/kg/day (group 6), respectively. Low doses of each of the antiresorptive drugs (1/10 of optimal concentration), A (Low; group 5) and R (Low; group 7), were also tested to establish the dose-dependent effect for each drug. For groups 8 to 11, the combined treatment of ELP with high or low dose of A or R was set up in order to study the interactions between each selected herb-drug pair.

### 2.4. Bone Mineral Density Determination

Changes in bone mineral density (BMD) at lumbar vertebra (L5), proximal tibial metaphyses, and distal femoral metaphyses of the rats were monitored using a peripheral quantitative computed tomography (pQCT) (XCT2000, Stratec Medizintechnik, GmbH) bi-weekly within the eight weeks of the experimental period. The day on which treatment started was Day 0. The coefficient of variation (CV%) of standard measurements was less than 3%. First, the rat was anesthetized using a cocktail of ketamine and xylazine (100 mg/kg body weight and 10 mg/kg body weight, respectively) intramuscularly. It was then fixed on a custom-made translucent plastic holder. Lumbar spine (L5), right proximal tibia, and distal femurs were scanned under the built-in research mode of the pQCT. The scan speed was 25 mm/sec with voxel resolution 0.2 mm. Total BMD (BMD including both cortical and trabecular areas) was generated and presented.

### 2.5. Bone Microarchitectural Analysis

The microarchitecture of the left distal femur was analyzed using a microCT (Micro CT 40, Scanco Medical, Switzerland) after the rats had been euthanized. Briefly, the femur was aligned perpendicularly to the scanning axis. The scanning was conducted at 55 kVp and 144 *μ*A with a resolution of 16 *μ*m per voxel. The trabecular bone within the distal femur was identified with semiautomatically drawn contour at each two-dimensional (2D) sections. Segmentation parameters were fixed at: Sigma = 0.5, Support = 1.0, and Threshold = 245. The volume of interest (VOI) was determined within 50 continuous slices. The microarchitectural parameters of the VOI were obtained through three-dimensional reconstructed images with the image analysis program of the micro-CT workstation. Parameters from direct model (bone volume density (BV/TV), trabecular number (Tb.N), trabecular thickness (Tb.Th), and trabecular plate separation (Tb.Sp)) were analyzed.

### 2.6. Biomechanical Test

Right femur was harvested after the rat had been sacrificed. It was wrapped with 0.9% saline-soaked gauze and placed in a resealable plastic bag immediately. All samples were stored at −20°C. Overnight thawing at room temperature (24°C) of the specimen was allowed before biomechanical test. Three-point bending test was performed using Hounsfield material testing machine (KM25, Redhill, UK). A load cell with maximum 250 N was mounted. The span of the lower supports was 20 mm. The midshaft of the bone was loaded at a constant speed of 5 mm/min in medial-lateral approach until failure. Strengths at yield, maximum, and break were recorded for analysis.

### 2.7. Serum and Urinary Biochemical Markers Determination

Serum osteocalcin (OC) concentration was assayed using rat OC ELISA kit from Biomedical Technologies (USA). Urinary level of deoxypyridinoline cross-links (DPD) was determined by ELISA kit (Quidel, USA). A standard curve was generated from each kit and the concentrations were calculated from the standard curves.

### 2.8. Statistical Analysis

The differences between treatments and control groups were tested with either (i) one-way analysis of variance (ANOVA) or (ii) Kruskal-Wallis test, followed by the Posthoc Dunnet's or Dunn's test, respectively, depending on the data distribution. The groups with alendronate and raloxifene were compared separately. All the covariates were adjusted for the statistical analysis. All statistical analyses were performed by using the Statistical Package of Social Science (SPSS) version 15.0 for Windows and carried out at the 5% level of significance (*P* < 0.05). Data were expressed as mean ± standard error of the mean (SEM).

## 3. Results

### 3.1. Chemical Characterization of ELP Extract

**Figure 1:**
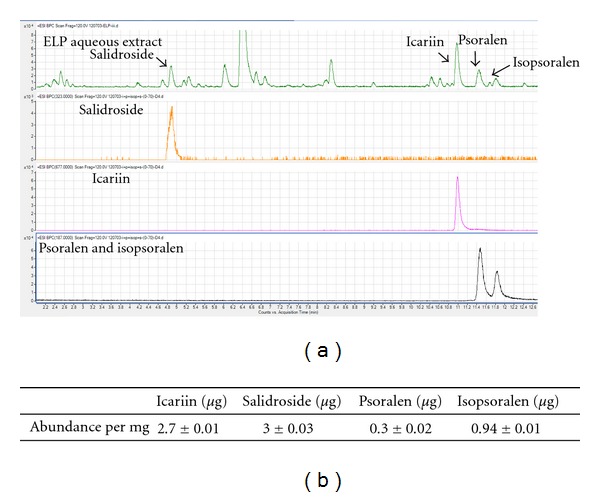
(a) Comparison of LC-MS base peak chromatograms from ELP aqueous extract and standard chemical markers, salidroside, icariin, psoralen, and isopsoralen; (b) Quantitative analysis of each marker in ELP aqueous extract.

The compositions of marker compounds in ELP aqueous extracts as determined by LC-MS were shown in Figure 1. The retention time of salidroside, icariin, psoralen, and isopsoralen were 4.68, 10.99, 11.45, and 11.81 min, respectively (Figure 1(a)). Isomers psoralen and isopsoralen were well separated using this elution condition. The presence of all marker compounds revealed the presence of Epimedii Herba, Ligustri Lucidi Fructus, and Psoraleae Fructus in ELP extract after a series of preparation processes. In the extract sample, salidroside was the most abundant, followed by icariin, and isopsoralen and psoralen (Figure 1(b)).

### 3.2. Model Establishment and Body Weight

**Figure 2:**
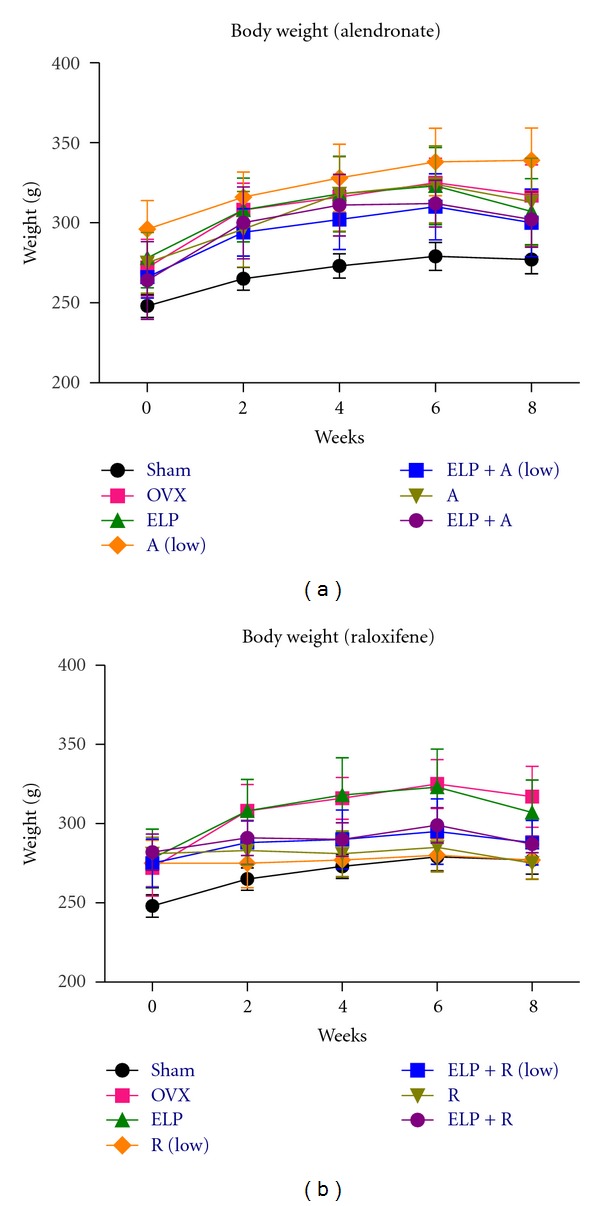
Mean of rat body weight between week 0 (baseline) and week 8. Rat body weight with different treatment was illustrated: (a) with alendronate; and (b) with raloxifene. The error bar represents the SEM for each treatment group (*n*  = 8 per group).

For the model establishment, ovariectomy caused a significant decrease of total BMD in lumbar spine, femur, and tibia by 6.5 ± 2.7%, 9.65 ± 3.0%, and 14.37 ± 3.4%, respectively, after three weeks of pretreatment period. During the next 8 weeks of treatment, we found that different combinations of ELP, A, and R did not cause significant change of rat body weight when compared with the OVX without treatment as shown in Figures 2(a) and 2(b).

### 3.3. Total BMD Analysis

**Figure 3:**
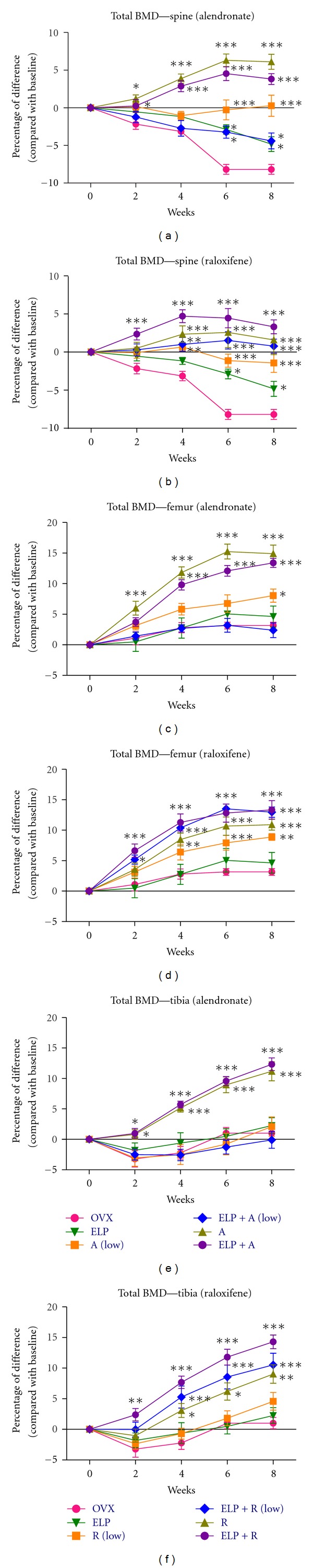
Mean of percentage difference of total BMD in lumbar spine, distal femur, and proximal tibia between week 0 (baseline) and week 8. BMD changes at different regions with different treatment, were illustrated: (a) lumbar spine with alendronate; (b) lumbar spine with raloxifene; (c) distal femur with alendronate; (d) distal femur with raloxifene; (e) proximal tibia with alendronate; (f) proximal tibia with raloxifene. The error bar represents the SEM. Significant difference: *P < 0.05; **P < 0.01; ***P < 0.001 for difference from OVX group without treatment at the corresponding time point.

At lumbar spine of OVX group, the effect of ovariectomy in lowering the total BMD was prominent. The total BMD decreased continuously from the baseline (Day 0) to 8.2% during the 8 weeks of treatment period (Figure 3(a)). However, the total BMD of distal femur and proximal tibia was not varied from the baseline (Figures 3(c) and 3(e)). This observation may indicate that ovariectomy results in a longer BMD reduction period in nonweight bearing bone (i.e., lumbar spine) than in weight bearing bones (i.e., femur and tibia) in ovariectomized rats. For the sham group, overall increase in total BMD was observed in all studied regions from baseline to 6.0% (lumbar spine), 11.07% (distal femur), and 14.16 (proximal tibia) at week 8 (data not shown).

All the 9 treatment groups had a higher BMD than OVX significantly at the lumbar spine from week 6. ELP significantly reduced the BMD loss by 5.3% (at week 6; *P* < 0.001) and 3.4% (at week 8; *P* = 0.011) when compared with corresponding OVX groups (Figure 3(a)). This finding was in line with our previous report [10]. However, no significant difference was observed throughout the experiment when ELP was compared with OVX at both distal femur and proximal tibia (Figures 3(c) and 3(e)). This observation reveals that ELP was more effective in prevention of bone loss in nonweight bearing bones than in increasing bone gain in weight bearing bones. For the antiresorptive drugs, dose-dependent effects of A and R were observed at all lumbar spine, distal femur, and proximal tibia. This effect has also been reported in other animal studies [17–19]. Both A (Figures 3(a), 3(c), and 3(e)) and R (Figures 3(b), 3(d), and 3(f)) at their optimal dose could raise the total BMD of the osteopenic rats significantly in all the 3 regions when compared with OVX. The protection efficacies of A were always higher than those of R in all studied regions at optimal dose.

For the combination studies, our data demonstrated that ELP extract could work synergistically with raloxifene in increasing BMD of osteopenic bone. When the osteopenic rats were treated with 0.25 mg/kg/day raloxifene, R (Low), a significant reduction of total BMD loss in lumbar spine and distal femur was observed, but with the lessened efficacies to its optimal dose from week 4 onwards. However, R (Low) had no significant effect in reducing total BMD loss at proximal tibia, similar to ELP group. When the osteopenic rats were cotreated with ELP and R (Low), significant increase in total BMD was observed at femur (Figure 3(d)) and tibia (Figure 3(f)) starting from week 2 to week 8, versus OVX. Interestingly, the bone protective effect of ELP + R (Low) was better than that of R alone at optimal dose from week 2 onwards. Similar observation was found in the lumbar spine, ELP + R (Low) totally abolished the decrease of BMD due to ovariectomy (Figure 3(b)). Cotreatment of ELP and R (Low) even led to higher BMD than R in the weight bearing bones throughout the experiment. Besides, dose-dependent effects of ELP + R and ELP + R (Low) were observed at lumbar spine and tibia. Coadministration of ELP and *R* increased the total tibial BMD by 5.26% (2.5 mg/kg/day of R; *P* = 0.014) and 5.94% (0.25 mg/kg/day of R; *P* = 0.026) when compared with the respective dosage groups with R alone. ELP + R was the most effective treatment group among those groups with R or ELP extract alone in spine and proximal tibia. However, such synergistic effect was absent in all the groups cotreated with ELP and alendronate.

### 3.4. Bone Microarchitecture Analysis

**Figure 4:**
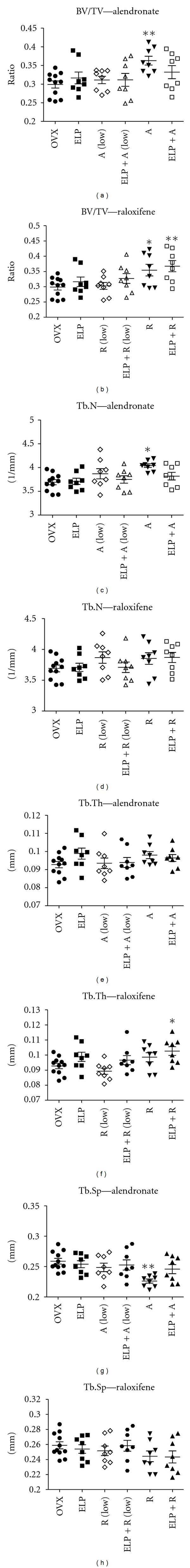
Alterations in trabecular bone volume (BV/TV), trabecular number (Tb.N), trabecular thickness (Tb.Th), and trabecular separation (Tb.Sp) at the distal femur metaphysis following 8 weeks of treatment with different combinations of alendronate (a, c, e, and g) and raloxifene (b, d, f, and h). Bars represent the mean ± SEM for each treatment group (*n*  = 8 per group). Significant difference: *P < 0.05; **P < 0.01 for difference from OVX group without treatment.

Although total BMD has been long regarded as a surrogate measure of bone strength, microarchitectural properties provide more comprehensive information to evaluate the impact of different combinations on the quality of femoral trabecular bone. All the treatment groups showed a trend of improvement in the microarchitectural properties of the trabecular bone, including higher bone volume fraction (BV/TV), trabecular number (Tb.N), and trabecular thickness (Tb.Th), but lower trabecular separation (Tb.Sp) than OVX. ELP treatment showed a mild, but insignificant increase in BV/TV, Tb.N, and Tb.Th and decrease in Tb.Sp, which was directly correlated with the insignificant increase of total BMD in femoral region, as shown in Figure 3(c). In contrast, a dose-dependent effect of the A and R in increasing bone volume at distal femur of the osteopenic rats was observed. The rats treated with A had a higher BV/TV (*P* = 0.007; Figure 4(a)), Tb.N (*P* = 0.004; Figure 4(c)) but lower Tb.Sp (*P* = 0.002; Figure 4(g)) than OVX. For Tb.N and Tb.Sp, significant differences were not observed in R-treated groups (Figures 4(d) and 4(h)).

Similar to the total BMD analysis, ELP extract could synergistically enhance the efficacy of R at all doses in ovariectomized rats. However, such synergistic effect was absent in all groups cotreated with A. When the rats were cotreated with ELP and R (Low), there was an increasing trend in BV/TV (Figure 4(b)) and Tb.Th (Figure 4(f)), but statistical significance was not achieved, likely due to lower magnitude of changes and limited repetitive tests performed. At higher concentrations of R, ELP + R simultaneously had a further improvement in the microarchitectural properties compared with those treated with R alone. Significant difference was found in BV/TV (*P* = 0.007; Figure 4(b)) and Tb.Th (*P* = 0.038; Figure 4(f)), when compared with OVX groups.

### 3.5. Biomechanical Testing

**Figure 5:**
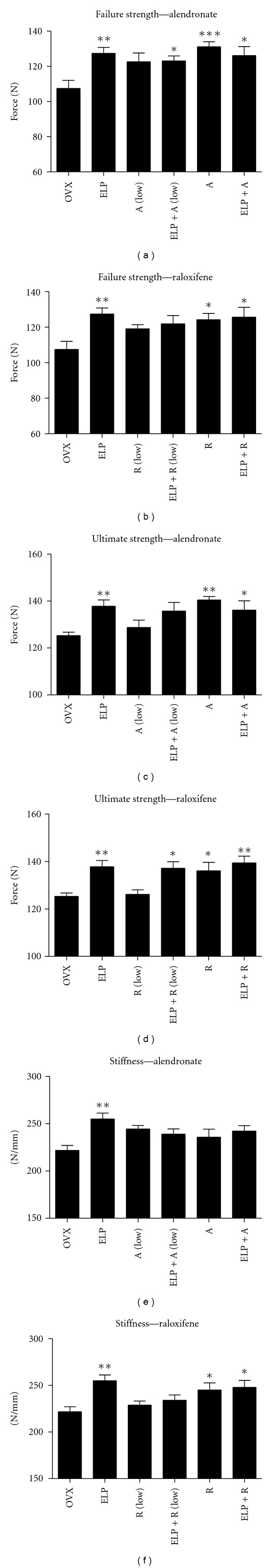
Alterations in failure strength, ultimate strength and stiffness at the femoral midshaft following 8 weeks of treatment with different combinations of alendronate (a, c, and e) and raloxifene (b, d, and f). Bars represent the mean ± SEM for each treatment group (*n*  = 8 per group). Significant difference: *P < 0.05; **P < 0.01; ***P < 0.001 for difference from OVX group without treatment.

Our data on mechanical testing of bones showed that all groups treated with ELP, alendronate, or raloxifene at optimal dose increased the biomechanical properties of the mid-shaft femur of osteopenic rats (Figure 5). Both ELP and R increased the failure strength, ultimate strength, and stiffness significantly compared with OVX, while A increased failure and ultimate strengths significantly. Dose-dependent effect of the two antiosteoporotic drugs on the biomechanical properties was also observed (except the stiffness by A).

For the combination studies, ELP extract could enhance the efficacy of R at all dose levels. On the contrary, such improvement only presented for the groups cotreated with low dose of A for the parameters in failure strength and ultimate strength. When the rats were cotreated with ELP and R (Low), there was an increasing tendency of failure strength (Figure 5(b)), ultimate strength (Figure 5(d)), and stiffness (Figure 5(f)). Similar observations were also found in those groups cotreated with ELP and R. These findings gave further evidence to support that ELP could enhance the function of R in a dose-dependent manner. However, the interaction between ELP and R is not considered as synergistic since the combined effects of any dose of R were weaker than ELP alone. This interaction is regarded as a simple additive.

### 3.6. Serum and Urinary Biochemical Markers Determination

**Figure 6:**
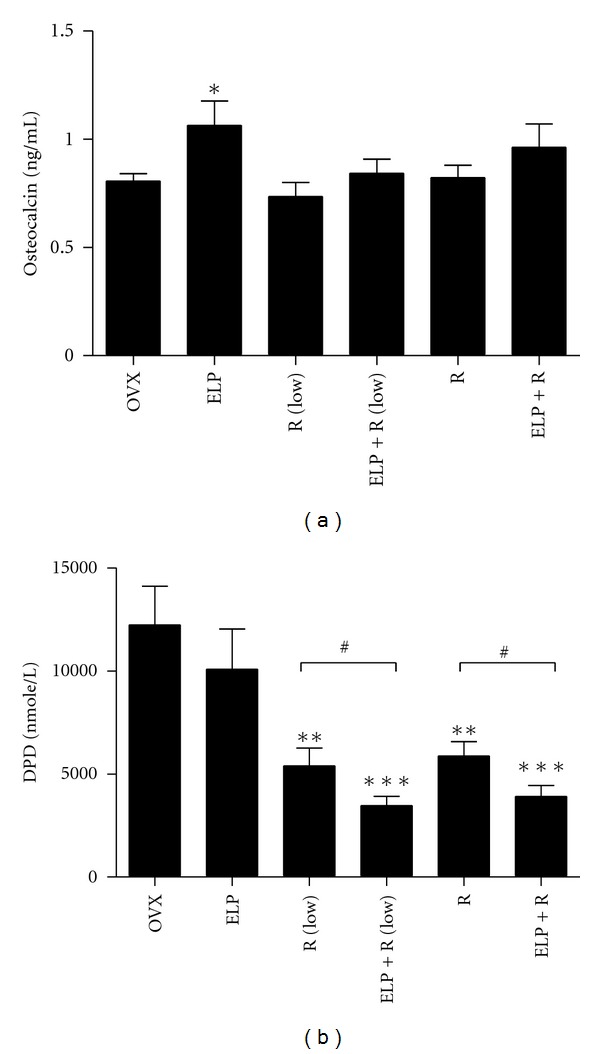
Effects of different combinations of raloxifene-related treatment in serum osteocalcin levels and excretory DPD levels after 8 weeks. Bars represent the mean ± SEM for each treatment group (*n*  = 8 per group). Significant difference: *P < 0.05; **P < 0.01; ***P < 0.001 for difference from OVX group without treatment at the corresponding time point. ^#^ *P* < 0.05 for difference from corresponding raloxifene group (at the same dosage) without ELP cotreatment.

In order to elucidate the synergistic effect of ELP and R on bone metabolism, the bone formation marker serum osteocalcin level and bone degradation marker urinary DPD level were analyzed.  Figure 6(a) showed the corresponding changes in serum osteocalcin level after 8 weeks of treatment. A significant increase in osteocalcin levels was observed in ELP treatment group (*P* = 0.031), but not in both R groups. Similar to the biomechanical test, ELP extract appears to enhance the efficacy of R at all dose levels, although did not reach the level of significance.  Figure 6(b) showed the corresponding changes in urinary DPD level after 8 weeks of treatment. Deoxypyridinoline crosslink (DPD), which represents structure degradation of bone by osteoclastic resorption, was effectively regulated by R at 0.25 mg/kg/day (*P* = 0.006) and 2.5 mg/kg/day (*P* < 0.001). There was a trend of decrease in DPD level in ELP treatment group. Interestingly, the coadministration of ELP with R could synergistically decrease the DPD level at both dosages significantly (R (Low), *P* = 0.0258; R, *P* = 0.0202), when compared to R alone. Collectively, these findings provided a proof of an important role for ELP in reinforcing the effects of R mainly through bone resorption inhibition and partially from bone formation enhancement.

## 4. Discussion

In our present study, composition of marker compounds in ELP aqueous extracts was determined by LC-MS. We showed that salidroside was the most abundant, followed by icariin, isopsoralen, and psoralen. It may be due to the fact that salidroside is highly soluble in water, while icariin, psoralen, and isopsoralen are water-insoluble in nature.

Comparing two antiresorptive drugs we used in the experiments, we found that alendronate was more effective than raloxifene in reducing overall total BMD loss. As shown in total BMD analysis, alendronate started to have the significant difference from OVX group from week 2, whereas raloxifene started from week 4. These findings were in line with those reported previously that alendronate has a higher efficacy than raloxifene in reducing the risk for osteoporotic fracture [20]. This finding also echoed with the results of bone microarchitecture analysis, which illustrated that the efficacy of alendronate to increase trabecular bone formation of osteopenic bone was better than that of raloxifene. It could also support a current clinical review which reported that onset of efficacy for nonvertebral fracture was reduced by 12 months by alendronate but was reduced by 36 months by raloxifene [21]. Significant reduction on hip fracture rate by alendronate, but not raloxifene, was also reported by Hopkins et al. [22]. However, for the combination studies, we found that ELP extract could work synergistically with raloxifene in increasing BMD of osteopenic bone. These findings were further substantiated by bone microarchitecture analysis and revealed that ELP had a synergistic effect with raloxifene, but not alendronate, in increasing the new bone deposition on trabecular surface. This observation will be further confirmed by histological analysis.

Interestingly, although ELP extract itself did not cause significant osteo-protective effect on material (BMD) and architectural (microarchitecture) properties of femur, it improved femoral cortical bone strength significantly as shown in the biomechanical test. This unmatched observation might be due to the fact that biomechanical properties of bone depend not only on material and architectural factors, but also the geometric parameters of cortical bone on mid-shaft of long bone [23]. Fracture load was correlated better with BSI (CSA) (a bone strength index including the cortical BMD and the cross-sectional area) of the bone than cortical BMD or total BMD alone in mid-shaft femur and humerus of goats. ELP might affect the geometry of the long bone cortex. Having not measured the geometric parameters was a limitation of this study.

Previously, it has already been demonstrated that ELP extract possessed beneficial effects in promoting bone health in aged ovariectomized rats [10], tail-suspended rats [9], and also in postmenopausal osteopenic women [11]. For the purpose of health supplement and/or pharmaceutical products development, the information related to the herb-drug interactions between ELP and standard antiresorptive drugs is necessary, since some of the osteopenic individuals may consume them simultaneously. In the present study, we have demonstrated that ELP extract itself was able to significantly reduce total BMD loss in lumbar spine and increase biomechanical strength of the bone and serum osteocalcin levels. Provided that ELP and antiresorptive drugs act on different molecular targets in bone metabolism, we postulate that synergistic effects could be generated when individuals take both together, so that the dosage of these antiresorptive drugs can be reduced. In this study, we found that ELP could selectively enhance the therapeutic effects of raloxifene, but not alendronate. In addition, ELP could enhance the effects of raloxifene even at 1/10 of optimal dosage.

To elaborate on the selectivity of ELP actions, we need to look into the characteristics of the animal models and also the fundamental working mechanisms of alendronate and raloxifene. An estrogen deficiency caused by ovariectomy results in (i) inhibition of mature osteoblasts and promotes faster osteoblastic apoptosis; and (ii) stimulation of osteoclast formation and bone resorption; eventually causes a net effect of reduced bone mass [24, 25]. The antiresorptive mechanism of alendronate and raloxifene is not the same. Alendronate is an inorganic pyrophosphate, which preferentially inhibits osteoclast-mediated bone resorption without affecting bone formation [26]. Raloxifene is an oral selective estrogen receptor modulator (SERM) that has estrogenic actions on bone and anti-estrogenic actions on the uterus and breast. It plays an analogous role to estrogens on bone tissue, and its osteoblastic action has recently been shown [27]. It was reported that raloxifene required osteoblastic cells to achieve its anti-osteoclastic action [28]. Previously, we found that ELP not only suppressed the osteoclast formation, but also enhanced the bone formation by increasing the osteogenesis of mesenchymal stem cells [9] and the proliferation of osteoblast as well (unpublished data). The additional osteoblastic actions from ELP may favor the anti-osteoclastic activities of raloxifene in inhibiting bone resorption, as supported by the decrease in DPD level (Figure 6(b)). It may offer a possible explanation as to why the presence of ELP always synergistically enhances the osteoprotective effects of raloxifene against estrogen deficient bone loss in all studied parameters. This suggestion will be verified using different other known anabolic agents, such as strontium ranelate or parathyroid hormone, in combination with raloxifene on the ovariectomized rats. Further experiments are also required to understand the basic underlying mechanisms behind the synergistic actions of ELP on raloxifene using monocytes/macrophages and osteoblast coculture system [28].

## 5. Conclusions

The primary goal of this work is to examine the drug interaction potential arising from an osteoprotective herbal formula, ELP. We found that ELP extract could (i) synergistically enhance the bone protective effects of raloxifene; and (ii) reduce the dose of raloxifene to achieve its biological effects. The coadministration of ELP may also minimize the adverse effects of raloxifene. The action of ELP was specific to raloxifene but not to alendronate. ELP may be developed as a novel complementary agent to those osteopenic individuals who are receiving raloxifene treatment. To the best of our knowledge, this is the first comprehensive study on the herb-drug interaction in osteoporosis management. These findings justify clinical studies using ELP and standard antiresorptive agents together in an attempt to counteract osteoporosis.

## Figures and Tables

**Table 1:** Grouping and treatment protocol.

Group	Description
(1) Sham	Sham-operated
(2) OVX	Ovariectomized (OVX)
(3) OVX + ELP	OVX treated with 0.35 g/kg/day ELP
(4) OVX + A	OVX treated with 0.5 mg/kg/day alendronate
(5) OVX + A (Low)	OVX treated with 0.05 mg/kg/day alendronate
(6) OVX + R	OVX treated with 2.5 mg/kg/day raloxifene
(7) OVX + R (Low)	OVX treated with 0.25 mg/kg/day raloxifene
(8) OVX + ELP + A	OVX treated with 0.35 g/kg/day ELP + 0.5 mg/kg/day alendronate
(9) OVX + ELP + A (Low)	OVX treated with 0.35 g/kg/day ELP + 0.05 mg/kg/day alendronate
(10) OVX + ELP + R	OVX treated with 0.35 g/kg/day ELP + 2.5 mg/kg/day raloxifene
(11) OVX + ELP + R (Low)	OVX treated with 0.35 g/kg/day ELP + 0.25 mg/kg/day raloxifene

## Abbreviations

ANOVA: One-way analysis of variance
R: Raloxifene
A: Alendronate
pQCT: Peripheral quantitative computed tomography
*μ*CT: Micro-computed tomography
LC/MS: Liquid chromatography-mass spectrometry
BMD: Bone Mineral density
BV/TV: Bone volume-to-tissue volume ratio
DPD: Deoxypyridinoline.
